# Examining Device Usage Patterns in Outpatient Telemedicine Video Visits among Movement Disorders Patients

**DOI:** 10.1002/mdc3.70275

**Published:** 2025-08-18

**Authors:** Mitra Afshari, Vijay G. Palakuzhy, Bichun Ouyang, Glenn T. Stebbins, Christopher G. Goetz

**Affiliations:** ^1^ Department of Neurology and Rehabilitation University of Illinois at Chicago Chicago Illinois USA; ^2^ Department of Neurological Sciences Rush University Medical Center Chicago Illinois USA

**Keywords:** Parkinson's disease, telemedicine, healthcare access, digital divide, healthcare disparities

## Abstract

**Background:**

Telemedicine has improved access to care, yet device usage for virtual visits remains underexplored.

**Objective:**

To characterize device usage patterns for video televisits across sociodemographic factors in a tertiary Movement Disorders clinic in the United States.

**Methods:**

We conducted a three‐year retrospective cross‐sectional analysis of 2181 video televisits, categorizing devices used as mobile (smartphones/tablets) or personal computers (desktops/laptops), and analyzed usage patterns across age, sex, race, and income.

**Results:**

Mobile devices were used in 88% of video televisits. Patients <60 years‐old preferred mobile devices over PCs (91%, *p* = 0.002). Lower income patients used only mobile devices. Significant differences were detected for age and income, but effect sizes were small. Logistic regression identified only age <60 as a predictor of mobile usage (OR 1.55, *p* = 0.0005).

**Conclusions:**

Mobile device usage for telemedicine is becoming increasing ubiquitous across sociodemographic factors, highlighting the need to optimize mobile telemedicine platforms and leverage mobile devices for teleresearch in Movement Disorders patients.

Telemedicine (TM) has transformed the landscape of healthcare delivery by overcoming traditional barriers to healthcare access, particularly during the SARS‐CoV‐2 pandemic.^1^, ^2^ For Movement Disorders in particular, there is more than a decade of evidence highlighting benefits of TM with respect to healthcare access and patient and carepartner burden, especially for the growing Parkinson's Disease (PD) population.^3^, ^4^, ^5^, ^6^, ^7^, ^8^, ^9^ While TM has clear potential to improve healthcare access among underserved populations, disparities related to socioeconomic status, digital literacy, and technology access, collectively known as the *digital divide*—continue to be questioned.^10^, ^11^, ^12^ Previous research has shown that older adults, lower‐income households, and individuals with limited digital literacy are less likely to utilize TM.^13^

Now that we are past the SARS‐CoV‐2 pandemic and the role of TM is being reevaluated, it is critical to investigate whether TM may actually act as a bridge for healthcare access due to increasing use of mobile devices like smartphones and tablets. In the past decade, mobile devices have seen dramatic growth, often replacing traditional personal computers (PCs) like laptops and desktops.^14^ For example, large cross‐sectional studies have revealed that a greater proportion of Black and Hispanic patients in the United States (US) use smartphones to access electronic health portals compared to their non‐Hispanic White and Asian counterparts.^15^, ^16^ Moreover, advances in the video and audio quality of mobile devices have made them more suitable for complex clinical interactions. In the US, governmental insurance coverage for telehealth services is still endangered, currently slated to expire in September 2025 unless new legislation is approved.^17^ Collectively, these historical developments emphasize the importance of understanding device usage patterns in TM to inform efforts towards its viability and expansion.

Our objective in this retrospective chart review was to examine device usage patterns (mobile vs PC) for outpatient video televisits across sociodemographic factors at a high‐volume tertiary Movement Disorders clinic in a large healthcare center serving a diverse population in the US. We hypothesized a high prevalence of mobile device usage across all sociodemographic factors.

## Methods

We conducted a three‐year retrospective chart review of video‐based TM encounters (“televisits”) at the Movement Disorders Clinic of Rush University Medical Center, a large tertiary care center located in the Chicago metropolitan area, serving urban‐, suburban‐, and rurally‐located patients from Illinois and the surrounding Midwest states insured by all major commercial and governmental plans in the US, including Medicare and Medicaid. The vast majority of patients seen in this clinic have Parkinson's Disease (≥75%). The study was approved by the Rush University Institutional Review Board. The final dataset was de‐identified and informed consent was waived as the study was retrospective and minimal risk to patients. The study included televisits conducted between March 2020 and March 2023 with 13 neurologists, three neuropsychologists, two psychiatrists, and two physician assistants who only see Movement Disorders patients.

Televisits were conducted through an electronic medical record (EMR)‐integrated, HIPAA‐compliant patient portal (Epic), accessible via either a mobile app or web browser. Throughout the study period, both access options for televisits were consistently available to patients without institutional preference or bias. The device used to access a televisit, identified from embedded metadata in the EMR, was categorized as either mobile (smartphone or tablet) or PC (desktop or laptop). We analyzed variations in device usage patterns for televisits across the following demographic variables: age at first encounter, sex, race, and socioeconomic status approximated using median household income based on patient ZIP code, sourced from the US Census Bureau, US Postal Service, and Internal Revenue Service records. Median household income was stratified into quintiles based on the 2022 US Census Income Classification.

Descriptive statistics were used to summarize demographic and device usage data. Chi‐square tests were used to evaluate distribution of device usage across demographic variables and effect sizes were calculated to quantify the strength of associations. A multivariable logistic regression model was constructed to evaluate the adjusted odds of mobile device usage, with covariates being age, sex, race, and income quintile. All statistical analyses were conducted using SAS software.

## Results

A total of 12,073 televisits from 3843 unique patients occurred over the three‐year period (Table 1). Of the 3843 unique patient encounters, 2181 were included in the final analysis after excluding televisits where device type could not be identified (*n* = 1434) and patients who used *both* device types during the study period (*n* = 228 patients). Across all demographic variables, the majority of televisits were conducted using mobile devices, with overall mobile usage comprising approximately 88% of all televisits.

**TABLE 1:** Demographic characteristics grouped by device type used to access televisits

Demographic category	Mobile users	PC users	Total	*p*‐value	Effect size
	*n* = 1915 (87.90%)	*n* = 266 (12.19%)	*n* = 2188		
Mean age at first encounter (SD)	62.15 (17.93)	64.86 (17.37)		0.02	0.15
Age at first encounter (*n*, %)				0.002	0.07
<60	618 (91.02)	61 (8.98)	679		
≥60	1297 (86.35)	205 (13.65)	1502		
Sex (*n*, %)				0.05	0.04
Male	906 (89.26)	109 (10.74)	1015		
Female	1009 (86.54)	157 (13.46)	1166		
Race (*n*, %)				0.08	0.06
White	1534 (87.01)	229 (12.99)	1763		
Black	139 (92.67)	11 (7.33)	150		
Asian	88 (92.63)	7 (7.37)	95		
Other	154 (89.02)	19 (10.98)	173		
Median household income (*n*, %)				0.03	0.07
Lowest quintile	46 (100)	0 (0)	46		
Second quintile	596 (88.80)	75 (11.18)	671		
Third quintile	1012 (87.47)	145 (12.53)	1157		
Fourth quintile	247 (84.59)	45 (15.41)	292		
Highest quintile	14 (93.33)	1 (6.67)	15		

*Note*: Percentages shown are the proportion of the total number of patients in each sociodemographic group.

### Age

**FIG. 1:**
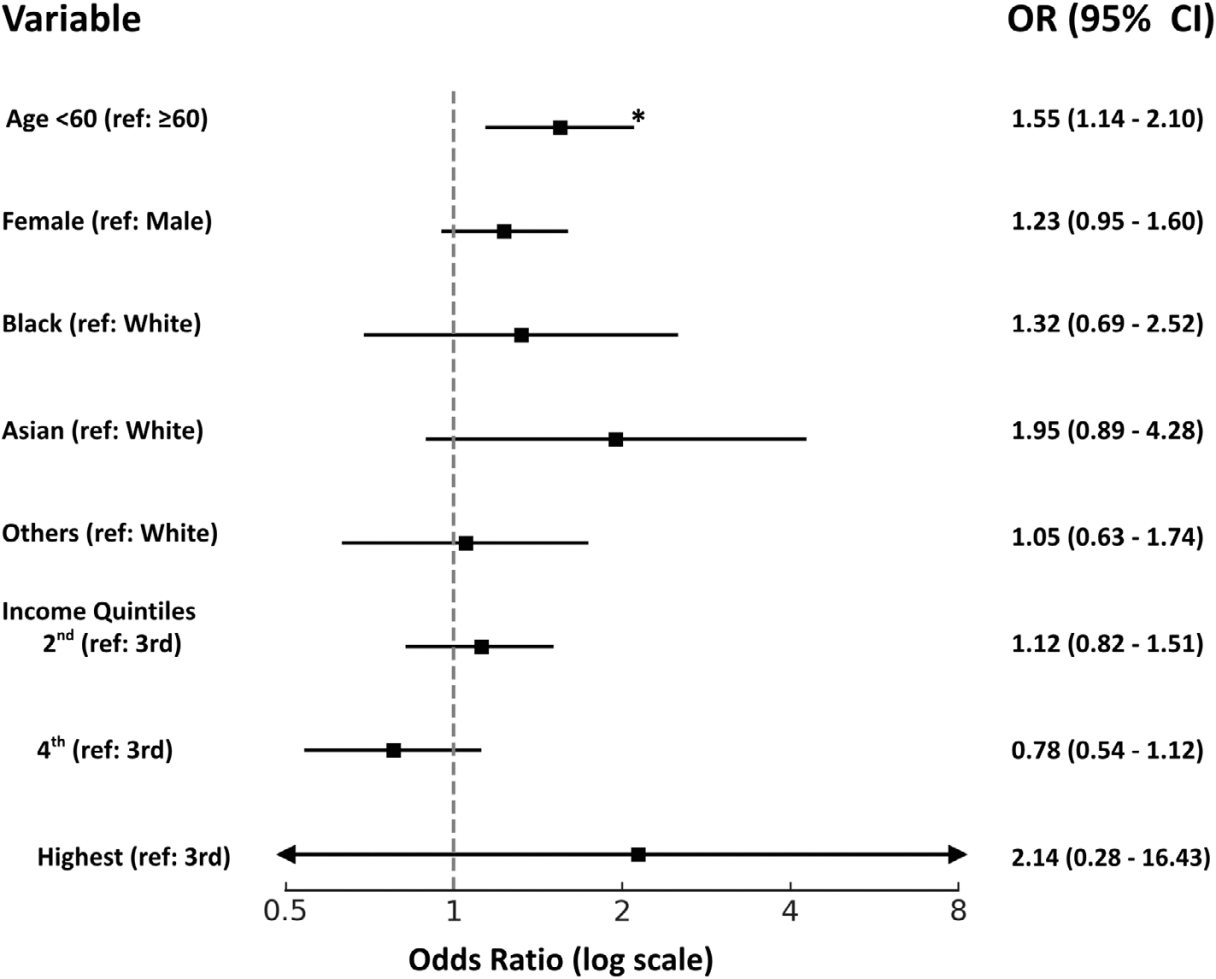
Forest plot illustrating the adjusted odd ratios for mobile device use for televisits. Asterisk indicates statistically significant results.

First, mobile device usage was more common among younger patients with the mean age of mobile device users being approximately 62 years, compared to 65 years among PC users (*p* = 0.02). Stratifying by age group with 60 years as the cutoff, approximately 91% of patients <60 years used mobile devices for their televisits (*p* = 0.002). There was a higher proportion of PC users in the ≥60 years group than in the <60 years group. However, the effect size for age was small (Cramer's *V* = 0.07). The logistic regression confirmed age <60 was a statistically significant independent predictor of mobile device usage, with an adjusted odds ratio (OR) of 1.55 (*p* = 0.0005) (Fig. 1). Age was the only variable that retained statistical significance in the logistic regression model.

### Sex

There was a slight female predominance in mobile device usage, but the difference was not statistically significant (*p* = 0.05). Sex did not emerge as a significant predictor of mobile device usage in the logistic regression model (OR 1.23, *p* = 0.12).

### Race

Mobile device usage was most prevalent among Black and Asian patients compared to White patients, however, these differences did not reach statistical significance in either the Chi‐square analysis or in the logistic regression model. While the adjusted ORs trended higher for mobile device usage among non‐White patients, these findings lacked statistical significance.

### Income

Device usage patterns varied across income quintiles and the differences were statistically significant (*p* = 0.03) though again the effect size was small (Cramer's *V* = 0.06). Notably, 100% of patients in the lowest income quintile used mobile devices for televisits, while patients in the fourth quintile had the highest relative preference for PC usage. In the logistic regression model, income quintile was not a significant predictor of mobile device usage.

## Discussion

To our knowledge, this is the first study that has formally investigated device usage for TM among Movement Disorders patients during the SARS‐CoV‐2 pandemic and beyond with specific attention to sociodemographic factors that potentially influence utilization. Our hypothesis was that mobile device usage would outweigh PC usage for televisits due to the increasing ubiquity of smartphone access across all populations, regardless of age, sex, race, and income. Our results confirmed our hypothesis, revealing that when Movement Disorders patients, a large proportion being older and having PD, were presented with the option of using a mobile device versus a PC for televisits, they overwhelmingly used mobile devices. In our analysis, statistically significant differences were associated with low effect sizes, indicating that although absolute numeric differences in device usage patterns were seen, the practical and clinical significance of these differences among various age, sex, race, and income groups were limited. This was owing to the large sample size and preponderance of mobile device usage. The multivariable logistic regression analysis showed that age <60 years as a predictor of mobile usage. Sex and race were not predictors of mobile usage despite higher usage among females, Black, and Asian patients. Lower income patients *only* used mobile devices, suggesting that mobile devices may be the preferred, primary, or only means of internet access for this group of patients. It is noteworthy that over 90% of patients (2181/2409) chose to use the same device type across multiple visits over the 3‐year period, suggesting consistent patient preference in device used for video encounters. While our cohort excluded patients who used both device types (*n* = 228 patients) to reduce analytic variability, future research could explore this subgroup to better understand preferences in different telemedicine settings. Moreover, future investigations could examine additional disease and visit characteristics such as provider type, diagnosis, disease severity, and complexity of the clinical encounter and their influence on device choice.

Overall, these findings suggest that mobile device usage and access to TM is becoming increasingly ubiquitous, even among our traditionally older Movement Disorders patients for whom we often question digital literacy. Our findings align with national trends showing increased “mobile‐first” health behavior among underserved groups with a significant proportion of low‐income adults in the US being “smartphone‐only” internet users.^18^ Previous research has shown that individuals in areas with limited broadband infrastructure and historically marginalized communities disproportionately access telehealth via mobile devices.^19^, ^20^ This supports the argument that mobile technology is not a workaround, but potentially a central solution to closing the *digital divide* and providing equitable care. It even raises the question of whether a *digital divide* still exists, or if this concern has become obsolete in this new era of advanced technology.

Furthermore, the dominance of mobile devices in our cohort may not only be a reflection of access but also a recognition of functionality, both for patients and Movement Disorders providers. Smartphones and tablets offer several advantages for virtual care—their portability enables convenience and easy repositioning for examinations, and many modern mobile devices feature higher‐resolution front‐facing cameras, improved audio capture, wider fields‐of‐view, and more recently, image stabilization that eliminates excessive camera motion (ie, tremor). There is strong evidence specifically in PD follow‐up care that TM may be equally efficacious when compared to in‐person care, owing to the largely visual examination.^21^, ^22^, ^23^, ^24^, ^25^, ^26^ A modified version of the MDS‐UPDRS Part III that can be captured remotely over video, without assessment of rigidity or postural stability, has demonstrated feasibility and preliminary validity against in‐person assessment, making it a viable remote outcome measure in decentralized research studies in PD.^22^, ^23^, ^24^, ^25^ Additionally, digital tools such as data from wearables, have enormous potential as objective outcome measures and may ultimately supplant remote MDS‐UPDRS assessments in PD teleresearch. Our findings support the critical need to shift our attention to optimizing TM platforms for mobile usage, monopolizing on engagement‐enhancing features like SMS messaging and “push” notifications, and developing novel health services using mobile devices.^27^ Establishing best practices is equally‐important as mobile devices are not necessarily designed for remote outcome assessments like the MDS‐UPDRS nor for Movement Disorders patients that struggle with cognitive impairment (ie, standardizing the virtual home environment with patient‐facing guides, requiring carepartner involvement).^28^, ^29^

Our study has several strengths, including a large sample of older adults over a three‐year period and analysis of real‐world device usage from embedded EMR data. We recognize that while this is a large cohort, the results are representative of only a single institution and its patient catchment with the majority of the patients being White. Other limitations include the exclusion of a large number of visits with undetermined device type, and lack of consideration of patient diagnosis, visit type, and potential provider influences on device choice.

## Author Roles

(1) Research project: A. Conception, B. Organization, C. Execution; (2) Statistical Analysis: A. Design, B. Execution, C. Review and Critique; (3) Manuscript Preparation: A. Writing of the first draft, B. Review and Critique.

M.A.: 1A, 1B, 1C, 2A, 2B, 2C, 3A, 3B.

V.G.P.: 1A, 1B, 1C, 2A, 2B, 2C, 3A, 3B.

B.O.: 2A, 2B, 2C.

G.T.S.: 1A, 1B, 1C, 2A, 2B, 2C, 3B.

C.G.G.: 1A, 1B, 1C, 2A, 2B, 2C, 3B.

## Disclosure

**Ethical Compliance Statement:** The study was approved by the Rush University Institutional Review Board (IRB) (ID: 23060505‐IRB01). The authors confirm that patient consent was not required for this work and therefore not obtained. We confirm that we have read the Journal's position on issues involved in ethical publication and affirm that this work is consistent with those guidelines.

**Funding Sources and Conflict of Interest:** This work was funded by a grant from the Rush University Department of Neurological Sciences awarded to MA. The authors declare that there are no financial disclosures or conflicts of interest relevant to this work.

**Financial Disclosures of All Authors (for the Preceding 12 Months):** MA has received research support from the Parkinson Study Group, Parkinson's Foundation, Consolidated Anti‐Aging Foundation (Chicago, Illinois, USA), and Rush University Department of Neurological Sciences, and consulting fees from Abbott, Inc. VGP reports no disclosures. BO reports no disclosures. GTS has received research support from the United States Critical Path Institute, Department of Defense, Dystonia Coalition, Cure Huntington's Disease Initiative, Michael J. Fox Foundation, International Parkinson and Movement Disorder Society, Ottawa Hospital Research Institute, University of California at San Diego, and University of California at San Francisco. GTS has received honoraria from the Alzheimer's Association, United States Critical Path Institute, International Parkinson and Movement Disorder Society, and Michael J. Fox Foundation. GTS has received consulting fees from the Cure Huntington's Disease Initiative Management, Huntington Study Group, Lily USA, Neurocrine Biosciences, Octave Bioscience, Pfizer, Vima Therapeutics, and WCG Clinical, Inc. CGG has received research support from the United States National Institutes of Health, National Institute of Diabetes and Digestive and Kidney Diseases, Department of Defense, and Michael J. Fox Foundation. CGG has received honoraria from the International Parkinson and Movement Disorder Society. CGG has received royalties from Elsevier Publishers, Wolters Kluwer, Oxford University Press, and Springer Science. CGG has received consulting fees from Theranova.

## Data Availability

The data that support the findings of this study are available upon request from the corresponding author. The data are not publicly available due to privacy.
